# Combined CD25-targeted near infrared photoimmunotherapy (NIR-PIT) and intratumor IL-15 enhance the effectiveness of anti-PD-1 immunotherapy

**DOI:** 10.1007/s00262-025-04186-x

**Published:** 2025-12-18

**Authors:** Makoto Kano, Aki Furusawa, Hiroshi Fukushima, Seiichiro Takao, Shuhei Okuyama, Hiroshi Yamamoto, Motofumi Suzuki, Miyu Kano, Ko Kitamura, Peter L. Choyke, Hisataka Kobayashi

**Affiliations:** Molecular Imaging Branch, Center for Cancer Research, National Cancer Institute, NIH, 10 Center Drive, Building 10, Room B3B47, Bethesda, MD 20892 USA

**Keywords:** Anti-PD-1 monoclonal antibody, CD8/regulatory T cell ratio, Hyperprogression, Interleukin-15, Near-infrared photoimmunotherapy

## Abstract

Immunotherapies aimed at enhancing anti-cancer immunity are now in wide clinical use. For instance, anti-programmed cell death protein 1 (PD-1) monoclonal antibody (mAb) therapy has demonstrated clinical efficacy in many tumor types, but overall, only a minority of patients respond. Near-infrared photoimmunotherapy (NIR-PIT) is a novel cancer treatment that selectively destroys targeted cells. CD25-targeted NIR-PIT is a strategy that selectively depletes regulatory T cells in the tumor microenvironment, thereby enhancing the T-cell response of PD-1 mAbs. Cytokines, such as interleukin-15 (IL-15), can also modulate immune response by further activating CD8^+^ T cells, thereby creating favorable CD8^+^/Treg ratio.

In this study, we evaluated the combination of CD25-targeted NIR-PIT and intratumoral IL-15 administration with anti-PD-1 mAb in treating syngeneic murine tumor models. The combination of CD25-targeted NIR-PIT and intratumoral IL-15 with anti-PD-1 mAb suppressed tumor growth, prolonged survival and increased the CD8^+^/Treg ratio. This combination achieved dramatic tumor control with 90% complete response and long-term survival, particularly in highly-immunogenic tumors. In addition, mice that achieved complete response resisted tumor re-inoculation suggesting durable memory T-cells were formed. In conclusion, the combination of CD25-targeted NIR-PIT and intratumoral IL-15 with anti-PD-1 therapy is a potentially useful strategy to improve the efficacy of immunotherapy.

**Supplementary Information:**

The online version contains supplementary material available at 10.1007/s00262-025-04186-x.

## Introduction

Immune checkpoint inhibitors have had a major impact on the treatment of many types of cancer and are now routinely used. However, only 15–30% of patients respond to immune checkpoint inhibitor therapy [1, 2], and some patients even experience hyperprogression or accelerated tumor growth after treatment [3]. Favorable tissue biomarkers of response include plentiful CD8^+^ T cell infiltration accompanied by relatively sparse regulatory T cells (Tregs), while an unfavorable tumor microenvironment (TME) consists of sparse CD8^+^ T cells and plentiful Tregs. Because a minority of patients have immune environments favoring response, attempts have been made to alter the TME prior to immune checkpoint inhibitor therapy [4–6].

Near-infrared photoimmunotherapy (NIR-PIT) is a relatively new anticancer therapy that uses a targeting monoclonal antibody (mAb) conjugated to a photoactivatable IRDye700DX (antibody-photo absorber conjugate, APC) and NIR light to eliminate cells expressing the cognate antigen of the mAb [7, 8]. Upon exposure to NIR light, the photochemical change in IR700 causes cell membrane damage only in the APC-bound cells. APC can be directed at a variety of membrane antigens found on tumors and stromal cells. For instance, anti-epidermal growth factor receptor (EGFR)-targeted NIR-PIT is approved in Japan for advanced head and neck cancers which typically express EGFR on cancer cells [9]. Tumor-targeted NIR-PIT not only directly destroys cancer cells but also promotes T-cell activation by inducing damage-associated molecular patterns release from the cancer cells, immunogenic cell death, and dendritic cell maturation with antigen presentation [10–12]. To expand the applicability of NIR-PIT to tumors lacking appropriate target antigens, such as EGFR, numerous preclinical studies have focused on targeting immunosuppressive cells within the TME [13, 14]. We previously reported that CD25-targeted NIR-PIT selectively depletes Tregs, leading to improved tumor responses. Immunosuppressive cell-targeted NIR-PIT enhances T-cell activation by relieving local immune suppression [13, 15].

Additionally, interleukin-15 (IL-15) is a cytokine that promotes the proliferation and activation of CD8^+^ T cells and natural killer (NK) cells [16–18]. It is thus a natural neoadjuvant for immunotherapies. We previously reported that intratumoral IL-15 administration in combination with tumor-targeted NIR-PIT enhances anti-tumor immunity while avoiding the side effects of systemic IL-15 administration [19]. Furthermore, the combination of CD25-targeted NIR-PIT and intraperitoneal injection of IL-15 induces an antitumor response in a tumor model [15].

We hypothesized that the combination of CD25-targeted NIR-PIT and intratumoral IL-15 would enhance the effects of anti-programmed cell death protein 1 (PD-1) mAb immunotherapy by improving the CD8^+^/Treg ratio. In this study, we assessed the efficacy of combining CD25-targeted NIR-PIT, intratumoral IL-15, and anti-PD-1 mAb in highly-immunogenic and poorly-immunogenic tumor models.

## Materials and methods

### Synthesis of IR700-conjugated anti-CD25-F(ab’)_2_ mAb

Anti-CD25-F(ab’)_2_ was generated from anti-CD25-IgG as described previously (6.8 nmol·L^−1^; clone PC-61.5.3; Bio X Cell, West Lebanon, NH, USA) [13]. APC was synthesized as described previously [20]. In this experiment, 1 mg of anti-CD25-F(ab’)_2_ mAb (9.1 nmol·L^−1^) was incubated with a three-point-seven-fold molar excess of IR700 NHS ester (10 mmol·L^−1^ in DMSO; LI-COR Biosciences, Lincoln, NE, USA). The resulting anti-CD25-F(ab’)_2_ mAb APC was designated as CD25-IR700.

### Tumor cell lines

Luciferase-expressing MC38 (murine colon cancer, MC38-Luc) cells were established from MC38 (RRID: CVCL_B288, American Type Culture Collection, Manassas, VA, USA, 2016) as previously described [13]. A murine oral cancer cell line, MOC2 (RRID CVCL_ZD33), was purchased from Kerafast (Boston, MA, USA) in 2020. MC38-Luc cells were cultured in RPMI 1640 medium (Thermo Fisher Scientific, Rockford, IL, USA) supplemented with 10% fetal bovine serum (FBS), 100 IU·mL^−1^ penicillin, and 100 µg·mL^−1^ streptomycin (Thermo Fisher Scientific) and MOC2 cells were cultured in the medium previously described [21], in a humidified incubator in an atmosphere of 95% air and 5% carbon dioxide at 37°C.

### Animal models

Six-week-old female C57BL/6 (strain #000664) mice were purchased from the Jackson Laboratory (Bar Harbor, ME, USA). The sample size was determined based on prior experience and power considerations to ensure adequate statistical validity. Each experimental group included 4 or 10 mice, resulting in a total of 16 or 40 mice. With 4 or 5 groups, the resulting E value (E = total number of animals − number of groups = 16 − 4 = 12, 20 − 5 = 15, or 40 − 4 = 36) exceeds the recommended minimum of 10, as suggested by NC3Rs and ARRIVE guidelines [22]. Two million MC38-Luc or MOC2 cells were subcutaneously injected into the right dorsum. The lower body of each mouse was shaved before treatment. Six days after cancer cell inoculation, mice were randomized using Tumor Manager (Biopticon, Princeton, NJ, USA) into separate treatment groups. Tumor volumes were determined by the following equation: volume (mm^3^) = length × width^2^ × 0.5 and were measured two or three times per week by caliper. The mice were euthanized when any of the following endpoints were reached: the tumor reached 2 cm in diameter, 2,000 mm^3^ in volume, showed severe ulceration, or had a ≥ 10% weight loss from baseline (Supplementary Fig. S1). The primary endpoints included tumor growth suppression, overall survival, and quantification of immune cell populations. Mice that survived 66 days after NIR-PIT and had no tumor were defined as a complete response (CR) for the purposes of this study.

### Intratumoral IL-15 administration

Human IL-15, obtained from the Biological Resources Branch (NCI-Frederick, Frederick, MD, USA), was administered intratumorally to mice (1 µg in 20 µL PBS) one hour after NIR-PIT, repeated every other day for 4 times.

### Anti-PD-1 mAb administration

Anti-mouse PD-1 (CD279) specific mAb (clone RMP1-14) was purchased from Bio X Cell. Anti-PD-1 mAb was administered to mice intraperitoneally starting one day before NIR-PIT and every other day thereafter for a total of 4 times. The first dose was 200 µg, and subsequent doses were 100 µg.

### In vivo NIR-PIT

During the procedure, the mice were anesthetized with inhaled isoflurane (2–3%) and intraperitoneal injection of ketamine (90 mg·kg^−1^) and xylazine (10 mg·kg^−1^). In the NIR-PIT treatment group, 100 µg of CD25-IR700 was injected via the retro-orbital vein 6 days after MC38-Luc cell or MOC2 cell inoculation, and NIR light (690 nm, 50 J/cm^2^) was applied to the tumor site. When NIR light was applied, the mice were shielded from light with aluminum foil, leaving only a small opening to expose the target tumor on the right dorsum.

Tumor-bearing mice were allocated into 4 groups as follows: (i) no treatment (Control group); (ii) IL-15 alone (IL-15 group); (iii) NIR-PIT alone (NIR-PIT group); (iv) the combination of IL-15 and NIR-PIT (Combination group). To assess the added benefit of anti-PD-1 mAb immunotherapy, tumor-bearing mice were randomized into two groups as follows: (i) a combination of IL-15 and NIR-PIT alone (Combination group); (ii) the combination of IL-15, NIR-PIT, and intraperitoneal anti-PD-1 mAb (Combination + anti-PD-1 group). To evaluate the efficacy of anti-PD-1 mAb monotherapy, tumor-bearing mice were randomized into two groups as follows: (i) no treatment (Control group); (ii) intraperitoneal anti-PD-1 mAb administration (anti-PD-1 group). Each group consisted of 10 mice. To verify the APC accumulation and photoconversion, IR700 fluorescence images were acquired before and after NIR-PIT. For bioluminescence imaging (shown as photons/cm^2^/sr), D-luciferin (15 mg·mL^−1^, 200 µL; Gold Biotechnology, St. Louis, MO, USA) was injected intraperitoneally, and the mice were analyzed on a PRISM in vivo imaging system (MediLumine, Montreal, Canada) and Image J (NIH, Bethesda, MD, USA). Regions of Interest were set to include the entire tumor.

### Flow cytometry

To evaluate tumor-infiltrating lymphocytes (TILs), we harvested tumors 2 days after NIR-PIT (n = 4/group). Single-cell suspensions of tumors were prepared using the previously described protocol [21]. The cells were stained with the antibodies in Table 1. Dead cells were stained with Fixable Viability Dye (Thermo Fisher Scientific, Cat# 65-0865-14) and removed from the analysis. For intranuclear staining, cells were fixed and permeabilized using the Intracellular Fixation & Permeabilization Buffer Set (Thermo Fisher Scientific). The stained cells were analyzed on a FACSLyric (BD Biosciences, CA, USA) and evaluated using FlowJo software (FlowJo LLC). Cell types were determined as follows; CD8^+^ T cells: CD45^+^/CD3^+^/CD8^+^/CD4^−^, Tregs: CD45^+^/CD3^+^/CD4^+^/CD8^−^/FOXP3^+^, NK cells: CD45^+^/CD3^−^/NK1.1^+^, natural killer T cells: CD45^+^/CD3^+^/NK1.1^+^, dendritic cells: CD45^+^/(F4/80)^−^/CD11c^+^/(I-A/I-E)^+^, and macrophages: CD45^+^/Gr-1^−^/(F4/80)^+^. The gating strategy is shown in Supplementary Fig. S2. The weight of the excised tumors was determined, the number of single cells collected from the tumors was measured, and the number of immune cells per unit weight of the tumor was calculated. The ratio of CD8^+^/Treg was obtained by dividing their respective absolute number.

**Table 1:** Antibodies used in this study

*Flow cytometry*					
Antibody	Clone	Cat#	RRID	Dulation	Source
CD3e	145-2C11	100306	AB_312671	1:100	Biolegend
CD4	RM4-5	100559	AB_2562608	1:100	Biolegend
CD11c	N418	117306	AB_313775	1:100	Biolegend
CD25	3C7	101904	AB_312847	1:100	Biolegend
CD45	30-F11	103112	AB_312977	1:100	Biolegend
F4/80	BM8	123135	AB_2562622	1:100	Biolegend
Gr-1	RB6-8C5	108428	AB_893558	1:100	Biolegend
I-A/I-E	M5/114.15.2	107630	AB_2069376	1:500	Biolegend
NK1.1	PK136	108714	AB_389364	1:100	Biolegend
CD8a	53-6.7	45-0081-82	AB_1107004	1:100	Thermo Fisher Scientific
CD11b	M1/70	48-0112-82	AB_1582236	1:100	Thermo Fisher Scientific
FOXP3	FJK-16 s	48-5773-82	AB_1518812	1:100	Thermo Fisher Scientific

*Multiplex immunohistochemistry*					
Antibody	Clone	Cat#	RRID	Dulation	Source
CD8	EPR20305	ab209775	AB_2860566	1:500	Abcam
CD4	EPR19514	ab183685	AB_2686917	1:2000	Abcam
CD45	D3F8Q	70257S	AB_2799780	1:500	Cell Signaling Technology
FOXP3	1054C	101,904		1:2000	Novus Biologicals
pan-cytokeratin	rabbit poly	bs-1712R	AB_10855057	1:500	Bioss Antibodies

### Multiplex immunohistochemistry

To evaluate TILs distribution following each treatment, tumors were collected 4 days after NIR-PIT, and processed into formalin-fixed, paraffin-embedded sections (n = 4/group). Multiplex immunohistochemistry was conducted as previously reported, utilizing Opal Automation IHC Kit (Akoya Biosciences, Marlborough, MA, USA) and Bond RXm autostainer (Leica Biosystems, Wetzlar, Germany) [23]. Tissue sections were stained with 4’,6-diamidino-2-phenylindole (DAPI) and the antibodies listed in Table 1. Coverslips were applied with ProLong Diamond Antifade Mountant (Thermo Fisher Scientific). Stained slides were analyzed using Mantra Quantitative Pathology Workstation and inForm Tissue Finder software (Akoya Biosciences). The inForm software was trained to identify tissue types and cellular phenotypes based on the following criteria: pan-CK-positive areas were classified as tumor, while pan-CK–negative areas were defined as stroma. pan-CK^+^/CD45^−^/CD8^−^/CD4^−^  = cancer cells, pan-CK^−^/CD45^+^/CD8^+^/CD4^−^  = CD8^+^ T cells, and pan-CK^−^/CD45^+^/CD8^−^/CD4^+^/FOXP3^+^ = Tregs, respectively. Five representative images were acquired per tumor, and cell densities were calculated by integrating tissue area measurements with corresponding cell counts, and then averaging the results obtained from the five images. The ratio of CD8^+^/Tregs was obtained by dividing their respective densities within the tumor area.

### Tumor rechallenge

In the tumor rechallenge test, mice that achieved CR after combination therapy of IL-15 and NIR-PIT, as well as the combination therapy with the addition of anti-PD-1 mAb, were re-inoculated with two million MC38-Luc cells in the contralateral dorsum 75 days after NIR-PIT. As a control, tumor-naive C57BL/6 mice of the same age were inoculated with two million MC38-Luc cells.

### Statistical analysis

Data are presented as mean ± SEM. Statistical analysis was performed using GraphPad Prism 10.4.1 (GraphPad Software, La Jolla, CA, USA). Luciferase activity was analyzed by repeated measures two-way ANOVA followed by Tukey’s test for multiple-group comparisons, and by a mixed-effects model to evaluate the group × time interaction for two-group comparisons. Tumor volumes were compared using repeated measures two-way ANOVA. Post hoc Tukey’s test was applied for multiple group comparisons, whereas the group × time interaction was evaluated to assess overall differences in tumor progression for two-group comparisons. Survival was analyzed by Kaplan–Meier method, and the results were compared using the log-rank test with Bonferroni correction. For statistical analyses of immunological data, one-way ANOVA followed by Tukey’s test was used for comparisons among multiple groups, and unpaired *t*-test was applied for comparisons between two groups. Statistical significance was set at *p* < 0.05.

## Results

### Combined intratumoral IL-15 and CD25-targeted NIR-PIT is effective in vivo

**Fig. 1:**
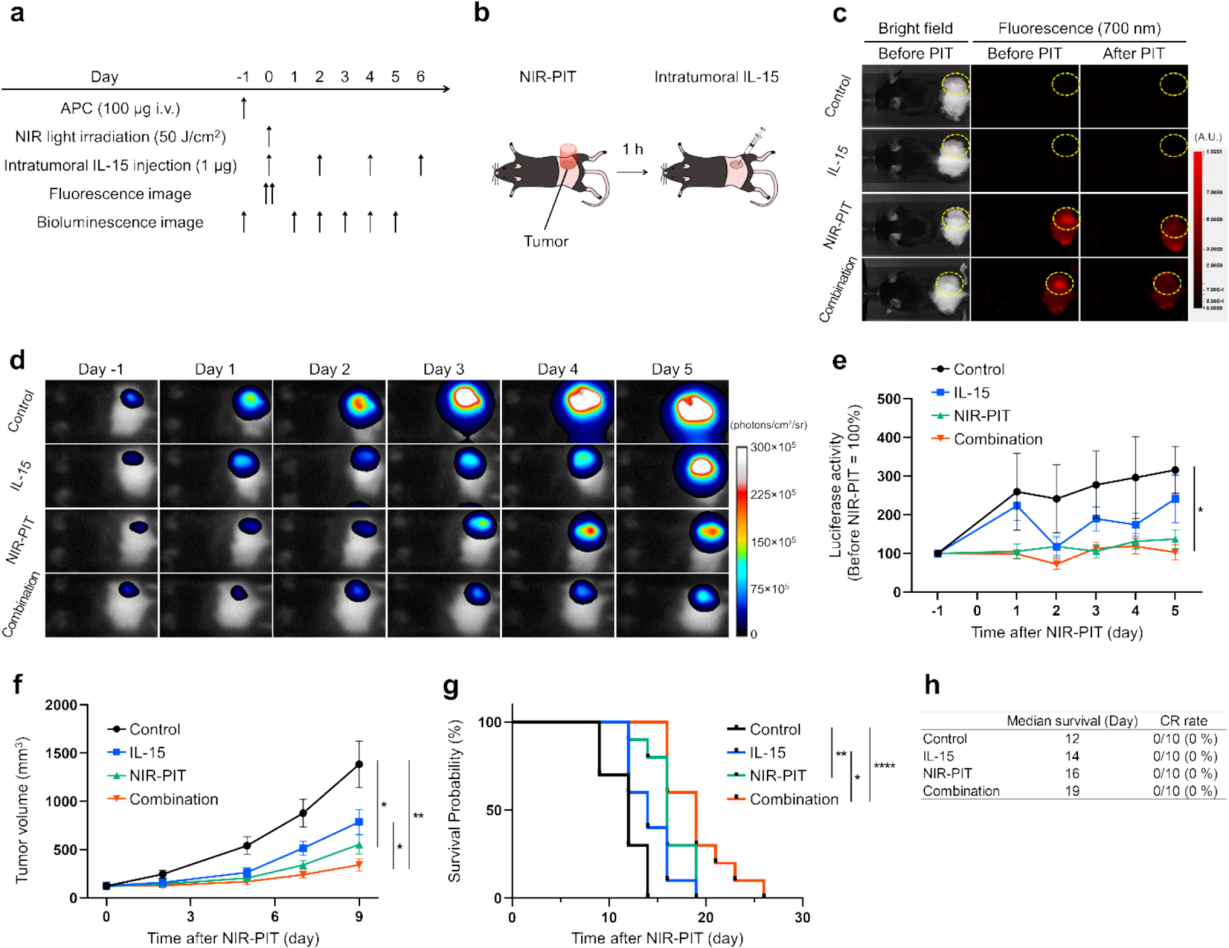
In vivo therapeutic effect of intratumoral IL-15 administration and CD25-targeted NIR-PIT for MC38-Luc tumor mouse model. Data was obtained from a single experiment. **a** Treatment schedule. **b** Diagram of treatment. Tumor was irradiated with NIR light, and, one hour later IL-15 was administered directly into the tumor. **c** Representative fluorescent imaging at 700 nm before and after NIR-PIT. Left white images: Bright-field images. Right two red images: 700 nm fluorescence images. Yellow dashed circles surround the tumor sites. **d** Representative bioluminescence imaging before and after NIR-PIT. **e** Assessment of luciferase activity measured using bioluminescence imaging before and after NIR-PIT (n = 10; mean ± SEM; repeated measures two-way ANOVA followed by Tukey’s test); *, *p* < 0.05 versus Control. **f** Tumor growth curves (n = 10; mean ± SEM; repeated measures two-way ANOVA followed by Tukey’s test); *, *p* < 0.05; **, *p* < 0.01; multiple comparisons. **g** Survival curves (n = 10, log-rank test with Bonferroni correction); *, *p* < 0.05; **, *p* < 0.01; ****, *p* < 0.0001; multiple comparisons. **h** Summary table of survival results

The therapeutic efficacy of the combination therapy of IL-15 and NIR-PIT was first evaluated using the highly-immunogenic MC38-Luc tumor model. The treatment schedule is shown in Fig. 1a, b. 700-nm fluorescent signals accumulated at tumor sites after APCs were administered. Those signals disappeared after NIR light irradiation, confirming IR700 photoactivation (Fig. 1c). Bioluminescence imaging was used to assess the cellular activity of cancer cells in the early stage of treatment. The luciferase activity in the Control group increased gradually, while that in the NIR-PIT and Combination groups was relatively reduced. Only the Combination group showed significantly suppressed cancer cell activity compared to the Control group (Fig. 1d, e). Figure 1f shows the tumor size within 4 different groups after treatment. The Combination group had the most tumor-growth suppression, followed by the NIR-PIT group. Survival was also the longest in the Combination group, and the second longest in the NIR-PIT group, but no animals achieved a CR (Fig. 1g, h). These results suggested that the intratumoral IL-15 and CD25targeted NIR-PIT combination therapy was effective but not curative in established tumor implants.

### Combination therapy of intratumoral IL-15 and CD25-targeted NIR-PIT significantly enhanced the CD8^+^/Treg ratio

To evaluate the early-stage response of immune cells within the TME induced by IL-15, NIR-PIT, or their combination, we measured immune cell populations in tumors 2 days after NIR-PIT.

**Fig. 2:**
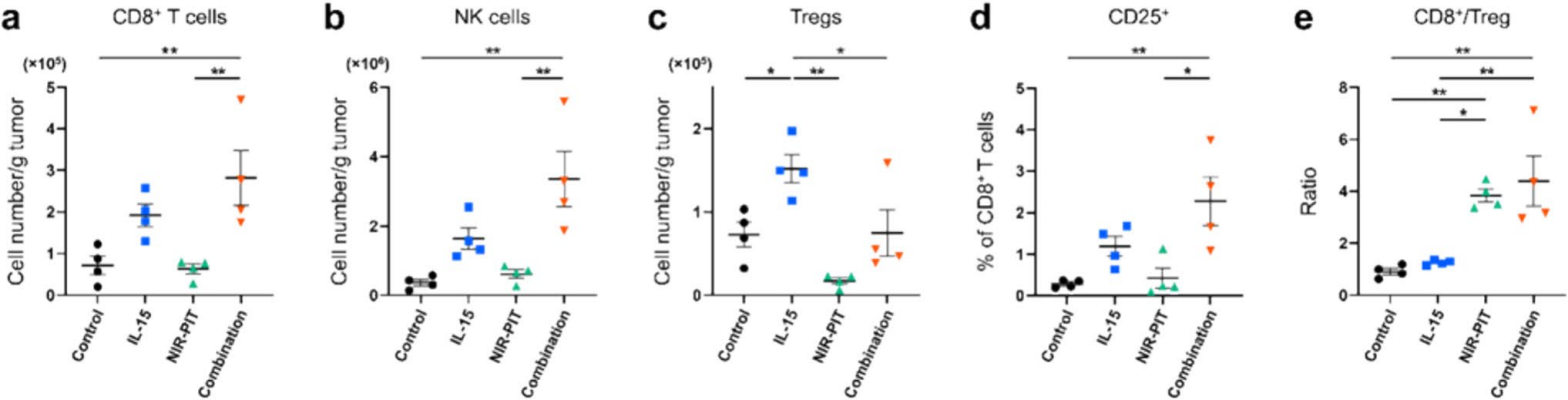
Effect of intratumoral IL-15 administration and CD25-targeted NIR-PIT on immune cells. Data was obtained from a single experiment. Immune cell populations within MC38-Luc tumor were analyzed by flow cytometry 2 days after CD25-targeted NIR-PIT. **a** **b** and **c**, the cell number/g tumor of **a** CD8^+^ T cells, **b** NK cells and **c** Tregs (n = 4; mean ± SEM; one-way ANOVA followed by Tukey test); *, *p* < 0.05; **, *p* < 0.01; multiple comparisons. **d** the percentage of cells expressing CD25 in CD8^+^ T cells (n = 4; mean ± SEM; one-way ANOVA followed by Tukey test); *, *p* < 0.05; **, *p* < 0.01; multiple comparisons. **e** the ratio of CD8^+^ T cells to Tregs is denoted CD8^+^/Treg (n = 4; mean ± SEM; one-way ANOVA followed by Tukey test); *, *p* < 0.05; **, *p* < 0.01; multiple comparisons

The number of CD8^+^ T cells and NK cells in the Combination group was significantly greater than that in the Control and NIR-PIT groups. A slight increase in the number of CD8^+^ T cells and NK cells was observed in the IL-15 group, although it was not statistically significant. (Fig. 2a, b). Comparable results were noted in natural killer T cells, dendritic cells, and macrophages (Supplementary Fig. S3). Conversely, IL-15 led to a significant increase in Tregs (Fig. 2c). Given that CD25 is expressed in both Tregs and activated T cells [24], we investigated whether NIR-PIT, which targets Tregs, adversely affected activated CD8^+^ T cells. There were very few CD25^+^ CD8^+^ T cells in the Control group, and the NIR-PIT did not significantly impact CD25^+^ CD8^+^ T cells. In contrast, the percentage of CD25^+^ CD8^+^ T cells was significantly higher in the Combination group suggesting activation of CD8^+^ T cells (Fig. 2d). Furthermore, the Combination group showed a significantly higher CD8^+^/Treg ratio, followed by the NIR-PIT group (Fig. 2e).

### The addition of anti-PD-1 mAb to intratumoral IL-15 and CD25-targeted NIR-PIT resulted in greater therapeutic efficacy

**Fig. 3:**
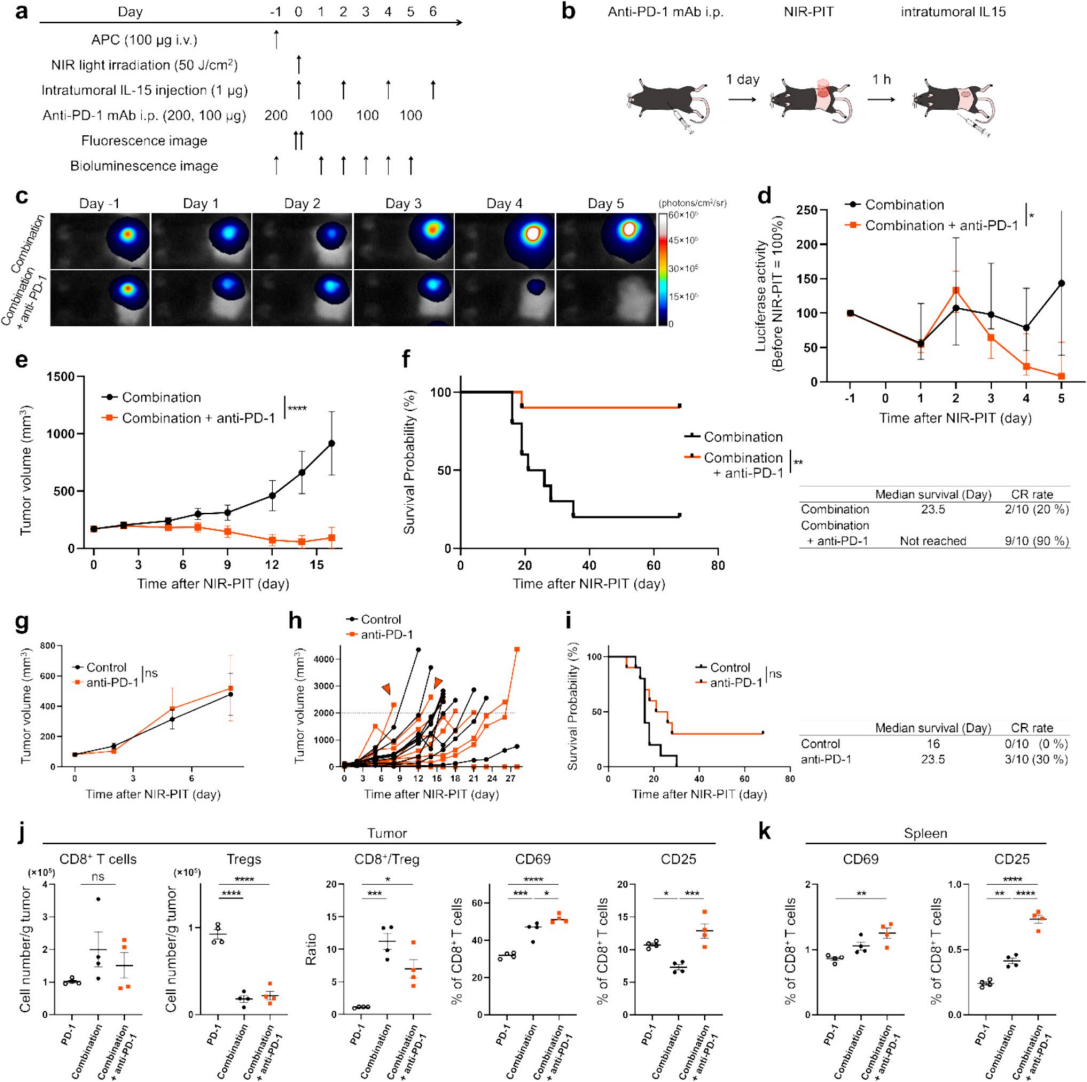
In vivo effect of anti-PD-1 mAb therapy after intratumoral IL-15 administration and CD25-targeted NIR-PIT in a highly-immunogenic MC38-Luc tumor mouse model. Data was obtained from a single experiment. **a** Treatment schedule. **b** Diagram of treatment. Anti-PD-1 mAb was administered intraperitoneally one day before NIR-PIT. Tumor was irradiated with NIR light, and IL-15 was administered directly into the tumor one hour later. **c** Representative bioluminescence imaging before and after NIR-PIT. **d** Assessment of luciferase activity measured using bioluminescence imaging before and after NIR-PIT (n = 8–10; mean ± SEM; Mixed-effects model focusing on the group × time interaction); *, *p* < 0.05. **e** Tumor growth curves (n = 10; mean ± SEM; repeated measures two-way ANOVA focusing on the group × time interaction); ****, *p* < 0.0001. **f** Survival curves and summary table of survival results (n = 10, log-rank test); **, *p* < 0.01. **g** Tumor growth curves (n = 10; mean ± SEM; repeated measures two-way ANOVA focusing on the group × time interaction); ns, not significant. **h** Individual tumor growth curves in the anti-PD-1 mAb therapy group and the Control group. Red arrowheads indicates cases in the anti-PD-1 mAb therapy group with more rapid growth than the Control group. The dashed line indicates a tumor volume of 2000 mm^3^. **i** Survival curves and summary table of survival results (n = 10, log-rank test); ns, not significant. **j** Immune cell populations within MC38-Luc tumor 2 days after CD25-targeted NIR-PIT. The two left indicate the cell number/g tumor of CD8^+^ T cells and Tregs, the third indicates the ratio of CD8^+^ T cells to Tregs, and the two right indicate the percentage of cells expressing CD69 and CD25 in CD8^+^ T cells (n = 4; mean ± SEM; one-way ANOVA followed by Tukey test); *, *p* < 0.05; ***, *p* < 0.001; ****, *p* < 0.0001; ns, not significant. **k** Immune cell populations within the spleen 2 days after CD25-targeted NIR-PIT. The percentage of cells expressing CD69 and CD25 in CD8^+^ T cells (n = 4; mean ± SEM; one-way ANOVA followed by Tukey test); **, *p* < 0.01; ****, *p* < 0.0001

We next evaluated the effect of adding anti-PD-1 mAb to the combination therapy using MC38-Luc tumor-bearing mice. Figures 3a, b show the treatment schedule. Fluorescent imaging at 700 nm confirmed that the APCs accumulated within the tumors, and this fluorescence disappeared after NIR light irradiation (Supplementary Fig. S4). Bioluminescence imaging showed a significant reduction in luciferase activity in the Combination + anti-PD-1 group compared to the Combination group without anti-PD-1 (Fig. 3c, d). Tumor growth was significantly inhibited (Fig. 3e), and survival was significantly prolonged, with a 90% CR rate in the Combination + anti-PD-1 group (Fig. 3f).

Anti-PD-1 mAb monotherapy was not effective in inhibiting tumor growth (Fig. 3g). The tumor growth rate in the anti-PD-1 group varied significantly, with some cases exhibiting extremely rapid growth akin to hyperprogression observed in some human patients, while others showed moderately decreased growth rates. (Fig. 3h, arrowhead). Anti-PD-1 mAb monotherapy achieved a 30% CR rate but did not demonstrate statistically significant prognostic improvement due to the variation in tumor growth rate (Fig. 3i).

The effect on immune cells caused by the addition of anti-PD-1 mAb to combination therapy among the anti-PD-1, Combination, and Combination + anti-PD-1 groups was examined by flow cytometry analysis 2 days after NIR-PIT. The increase of CD8^+^ was not significant, while Tregs were significantly reduced in the Combination and Combination + anti-PD-1 group compared to the anti-PD-1 group. As a result, the CD8^+^/Treg ratio in the two groups significantly increased compared to the anti-PD-1 group. In the Combination + anti-PD-1 group, the proportion of CD69-positive CD8^+^ T cells was significantly higher compared to the other two groups, and the proportion of CD25-positive CD8^+^ T cells was significantly higher compared to the Combination group (Fig. 3j). To evaluate the systemic immune response, the activation of T cells in the spleen was assessed 2 days after NIR-PIT. The percentage of CD69-positive CD8^+^ T cells was significantly higher in the Combination + anti-PD-1 group than in the anti-PD-1 group, while the percentage of CD25-positive CD8^+^ T cells was significantly higher in the Combination + anti-PD-1 group compared to both anti-PD-1 and Combination groups (Fig. 3k).

Collectively, these findings suggested that the combination therapy of IL-15 and NIR-PIT had an additive therapeutic effect to the anti-PD-1 mAb.

### Significant increase in CD8^+^/Treg ratio after combined intratumoral IL-15, CD25-targeted NIR-PIT, and anti-PD-1 mAb.

**Fig. 4:**
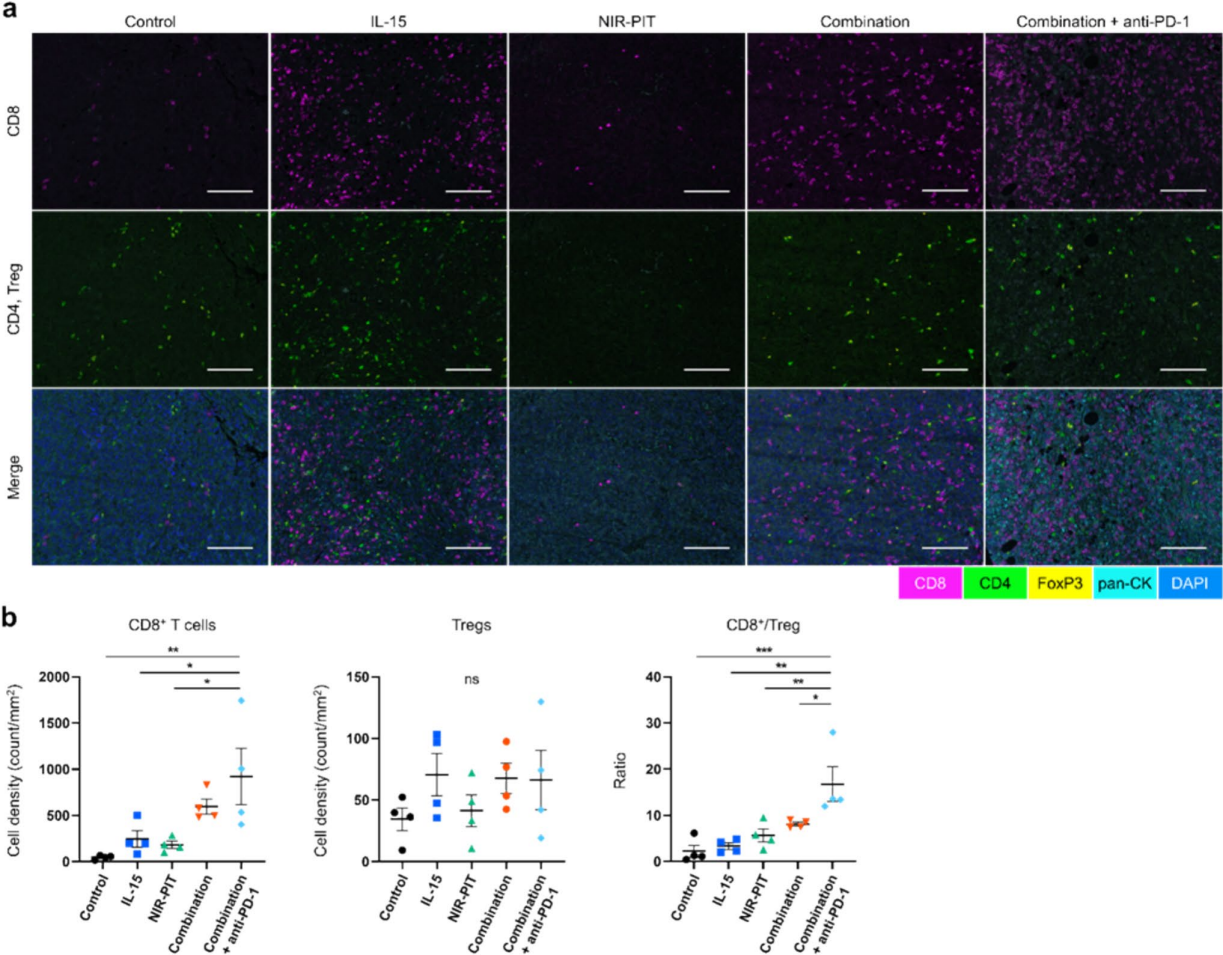
Assessment of tumor-infiltrating lymphocytes in a highly-immunogenic tumor mouse model. Data was obtained from a single experiment. Tumor-infiltrating lymphocytes were assessed by immunohistochemical analysis 4 days after CD25-targeted NIR-PIT in MC38-Luc model. **a** Representative multiplex immunohistochemistry images in MC38-Luc tumor. Antibody staining for CD8, CD4, FOXP3, pan-Cytokeratin (pan-CK), and DAPI are shown in magenta, green, yellow, cyan, and blue, respectively. **b** CD8^+^ T cell density (left), Tregs density (middle) and ratio of CD8^+^ T cells to Tregs (right) in tumor (n = 4; mean ± SEM; one-way ANOVA followed by Tukey test); *, *p* < 0.05; **, *p* < 0.01; ***, *p* < 0.001; ns, not significant; multiple comparisons

To evaluate the status of TILs after combination (IL-15, NIR-PIT) therapy and anti-PD-1 therapy, we analyzed the CD8^+^ T cells and Treg distribution inside the tumor tissue 4 days after NIR-PIT using immunohistochemical analysis. T cell distributions were compared across five groups: Control, IL-15, NIR-PIT, Combination, and Combination + anti-PD-1. All treatment interventions increased CD8^+^ T cell density in the tumor area, with only the Combination + anti-PD-1 group showing a significant increase compared to the Control, IL-15, and NIR-PIT groups (Fig. 4a, b left). There was no significant change in Treg count (Fig. 4, b middle). The CD8^+^/Treg ratio in the Combination + anti-PD-1 group was significantly higher than that of the other 4 groups (Fig. 4b right). These results showed that Combination + anti-PD-1 therapy promoted CD8^+^ T cell dominance among TILs.

### Long-lasting anti-tumor immune memory was induced by intratumoral IL-15 and CD25-targeted NIR-PIT with anti-PD-1 immunotherapy

To assess anti-tumor immune memory, MC38-Luc cells were re-inoculated into the mice 75 days after having previously achieved CR after combined IL-15, NIR-PIT, and anti-PD-1 mAb (n = 9, Supplementary Fig. S5) and combination therapy of IL-15 and NIR-PIT (n = 2, data not shown). All mice completely rejected the reinoculated MC38-Luc cells, while no graft rejection was observed in naive mice. These results revealed that anti-tumor immune memory was successfully established.

### Efficacy of intratumoral IL-15, CD25-targeted NIR-PIT, and anti-PD-1 mAb in poorly-immunogenic tumors

PD-1 blockade is not as effective in treating poorly immunogenic tumors characterized by low numbers of TILs and low tumor programmed death-ligand 1 expression [25]. To determine whether the combination therapy of IL-15 and NIR-PIT could modify poorly immunogenic TMEs and create a suitable environment for anti-PD-1 mAb therapy, the treatment was performed in mice bearing MOC2 tumors.

**Fig. 5:**
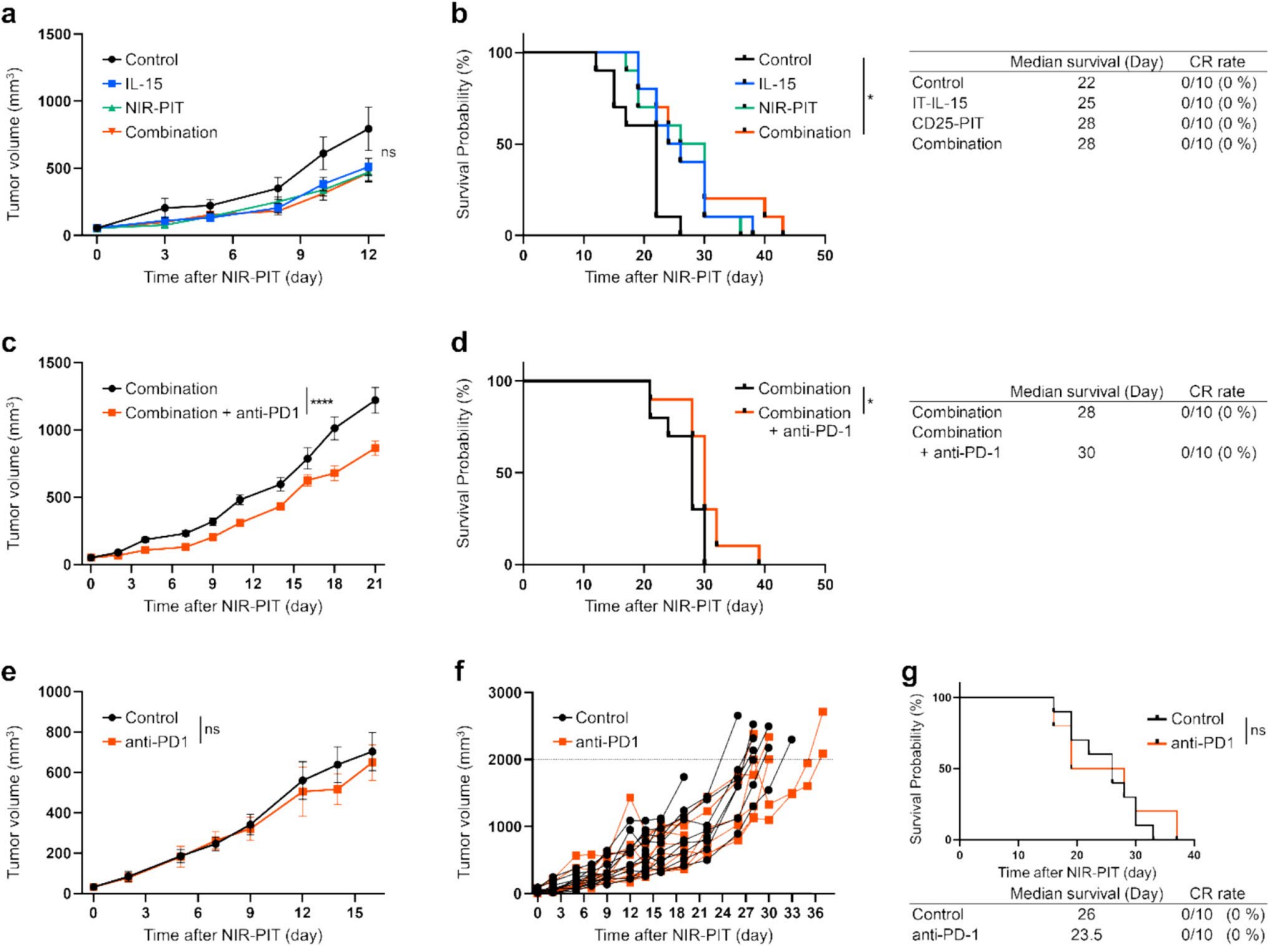
In vivo efficacy of intratumoral IL-15 administration and CD25-targeted NIR-PIT prior to anti-PD-1 mAb in a poorly-immunogenic MOC2 tumor mouse model. Data was obtained from a single experiment. **a** Tumor growth curves (n = 10; mean ± SEM; repeated measures two-way ANOVA followed by Tukey's test); ns, not significant; multiple comparisons. **b** Survival curves and summary table of survival results (n = 10, log-rank test with Bonferroni correction); *, *p* < 0.05; multiple comparisons. **c** Tumor growth curves (n = 10; mean ± SEM; repeated measures two-way ANOVA focusing on the group × time interaction); ****, *p* < 0.0001. **d** Survival curves and summary table of survival results (n = 10, log-rank test); *, *p* < 0.05. **e** Tumor growth curves (n = 10; mean ± SEM; repeated measures two-way ANOVA focusing on the group × time interaction). ns, not significant. **f** Individual tumor growth curves in the anti-PD-1 mAb therapy group and the Control group. The dashed line indicates a tumor volume of 2000 mm^3^. **g** Survival curves and summary table of survival results (n = 10, log-rank test). ns, not significant

We first examined the efficacy of IL-15 and NIR-PIT. The treatment schedule is shown in Supplementary Figure S6a. It was confirmed that APCs accumulated in the tumor and the fluorescence disappeared in response to NIR light (Supplementary Fig. S6b). Although there was no significant difference in tumor growth inhibition, the Combination group showed significantly prolonged survival compared to the Control group (Fig. 5a, b); however, no mice achieved CR.

Next, we investigated the efficacy of the addition of anti-PD-1 mAb to combination therapy. The treatment schedule is shown in Supplementary Fig. S6c. The accumulation of APC was confirmed by fluorescent imaging at 700 nm (Supplementary Fig. S6d). Significant inhibition of tumor growth and prolongation of survival were observed with the addition of anti-PD-1 mAb, although no mice achieved CR (Fig. 5c, d). Anti-PD-1 mAb therapy alone was ineffective in MOC2 tumors (Fig. 5e, f). The anti-PD-1 group did not have a significantly prolonged survival compared to the Control group (Fig. 5g).

These results indicated that the combination therapy of IL-15 and NIR-PIT facilitated anti-PD-1 mAb therapeutic efficacy in poorly-immunogenic tumors that were not responsive to anti-PD-1 mAb monotherapy.

### The CD8^+^/Treg ratio was increased by intratumoral IL-15, CD25-targeted NIR-PIT, and anti-PD-1 mAb in poorly-immunogenic tumors

To examine the effects of different treatments on immune cells in the MOC2 tumor model, flow cytometry and immunohistochemical analysis were performed 2 and 4 days after NIR-PIT, respectively.

**Fig. 6:**
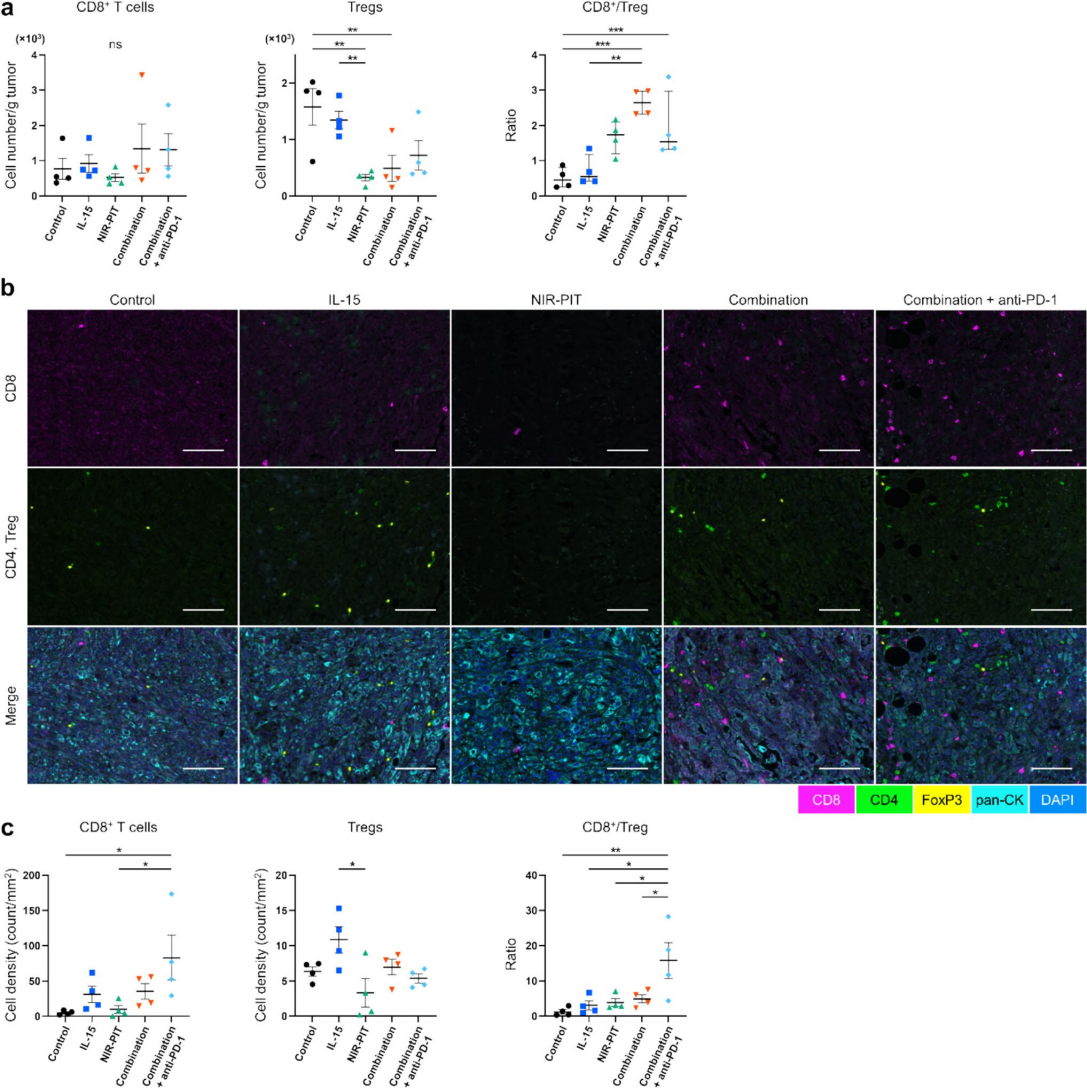
Assessment of tumor-infiltrating lymphocytes in poorly-immunogenic tumor mouse model. Data was obtained from a single experiment. Tumor-infiltrating lymphocytes were assessed by flow cytometry and immunohistochemical analysis 2 and 4 days after CD25-targeted NIR-PIT in the MOC2 model, respectively. **a** the cell number/g tumor of CD8^+^ T cells (left) and Tregs (middle) were evaluated by flow cytometry (n = 4; mean ± SEM; one-way ANOVA followed by Tukey test); **, *p* < 0.01; ns, not significant; multiple comparisons. The ratio of CD8^+^ T cells to Tregs is denoted as CD8^+^/Treg (n = 4; mean ± SEM; one-way ANOVA followed by Tukey test); **, *p* < 0.01; ***, *p* < 0.001; multiple comparisons. **b** Representative multiplex immunohistochemistry images in tumor of MOC2. Antibody staining for CD8, CD4, FOXP3, pan-Cytokeratin (pan-CK), and DAPI are shown in magenta, green, yellow, cyan, and blue, respectively. **c** Tumor-infiltrating lymphocytes were evaluated by immunohistochemical analysis. CD8^+^ T cell density (left), Tregs density (middle), and ratio of CD8^+^ T cells to Tregs (right) in tumor (n = 4; mean ± SEM; one-way ANOVA followed by Tukey test); *, *p* < 0.05; **, *p* < 0.01; multiple comparisons

Flow cytometry showed no significant change in CD8^+^ T cells across all the groups, while a significant reduction in Tregs in the NIR-PIT and Combination groups was observed, resulting in a high CD8^+^/Treg ratio in the two combination groups (the Combination group and Combination + anti-PD-1 group) compared to the Control group (Fig. 6a). Immunohistochemical analysis revealed that CD8^+^ T cells increased in the Combination + anti-PD-1 group compared to the Control and NIR-PIT groups (Fig. 6b, c left). Tregs were significantly higher in the IL-15 group than in the NIR-PIT group (Fig. 6b, c middle). The Combination + anti-PD-1 group produced the highest CD8^+^/Treg ratio (Fig. 6c right). These findings suggest that the combination of IL-15 and NIR-PIT created an optimal CD8^+^/Treg ratio in the early stages of immunotherapy, which was further augmented by subsequent anti-PD-1 mAb treatment.

## Discussion

This study investigated whether the combination of CD25-targeted NIR-PIT and intratumoral IL-15 could enhance the therapeutic efficacy of anti-PD-1 mAb therapy in two animal models, one with a highly-immunogenic TME and the other with a poorly-immunogenic TME. NIR-PIT and IL-15 showed a synergistic effect regardless of preexisting tumor immunogenicity (Figs. 1 and 5). The favorable CD8^+^/Treg ratio after the combination therapy was likely caused by IL-15 increasing the number of CD8^+^ T cells while NIR-PIT decreased the number of Tregs (Figs. 2, 4, and 6). Using this combination therapy with anti-PD-1 mAb immunotherapy resulted in significant additional benefits in both models, particularly in highly-immunogenic tumors (Figs. 3 and 5). Furthermore, mice who achieved a CR rejected tumor rechallenges, indicating the induction of long-term anti-tumor immunity (Supplementary Fig. S5).

IL-15 is a cytokine that belongs to the interleukin-2 (IL-2) family, and both IL-15 and IL-2 similarly promote the proliferation of CD8^+^ and NK cells. IL-15 is considered more suitable as an adjuvant for immunotherapy because IL-2 can cause the proliferation of Tregs which would be detrimental to the desired response [16–18, 26]. Nevertheless, IL-15 has also been reported to stimulate Tregs [27]. In this study, IL-15, in addition to increasing the number of CD8^+^ T cells, also increased Tregs, although it is unclear whether this was a direct effect of IL-15 or an indirect response to increased CD8^+^ T cells. However, IL-15 alone caused no change in the CD8^+^/Treg ratio. NIR-PIT directly kills Treg cells but also has the potential risk of depleting effector CD8^+^ T cells [24]. To mitigate this, we used anti-CD25-F(ab’)_2_ for NIR-PIT instead of IgG because it lacks an Fc region, avoiding Fc-mediated off-target effects, and is rapidly cleared from circulation, thereby minimizing the suppression of activated CD25⁺ T cells, ultimately contributing to superior antitumor efficacy [20]. Notably, the combination of NIR-PIT and IL-15 led to increases in the CD8^+^/Treg ratio and effector T cells, leading to a good therapeutic effect (Fig. 1e–h, Fig. 2d, e).

The CD8^+^/Treg ratio has been identified as a key determinant of the success of anti-PD-1 mAb therapy [6, 28]. The combination therapy of NIR-PIT and IL-15, by improving the CD8^+^/Treg ratio, set the stage for improved outcomes with anti-PD-1 mAb therapy. In parallel with the improvement of the CD8^+^/Treg ratio with NIR-PIT and IL-15, subsequent anti-PD-1 mAb therapy maintained the favorable CD8^+^/Treg ratio while effectively activating CD8^+^ T cells (Fig. 3j). Although Tregs gradually re-infiltrated the TME after treatment, the Combination + anti-PD-1 group exhibited an increase in CD8^+^ T cells without a corresponding significant increase in Tregs. As a result, the initially established favorable CD8^+^/Treg ratio was further amplified during the therapy (Fig. 4b), leading to a significant improvement in therapeutic outcomes (Fig. 3d–f). This therapy achieved a CR rate of 90% which significantly surpassed those previously reported with tumor-targeted PIT and intratumoral IL-15 administration [19] or CD25-targeted NIR-PIT and systemic IL-15 administration [15] despite the reduced dose of IL-15. Furthermore, comparing T cell activation in the spleens of the anti-PD-1 monotherapy and the Combination + anti-PD-1 group suggests that local tumor intervention also promotes systemic immune activation (Fig. 3k). Given the rejection of tumor rechallenges, the current combination local therapy is expected not only to achieve localized anti-tumor effects but also to enhance systemic immune responses and strengthen immune surveillance.

Hyperprogresion after immunotherapy is an ever-present concern. Hyperprogression has been associated with Treg-dominant TMEs [21, 29–31]. Some tumors showed rapid growth resembling hyperprogression in the anti-PD-1 mAb monotherapy group (Fig. 3h). These findings suggest that early modification of the TME to achieve a more favorable CD8^+^/Treg ratio by NIR-PIT and IL-15 could prevent anti-PD-1 mAb-induced hyperprogression and maximize therapeutic efficacy.

In poorly-immunogenic tumors that have fewer TILs, the immune checkpoint pathways do not strongly affect tumor growth [4]; so anti-PD-1 mAb alone showed limited effectiveness (Fig. 5e, g). There were no rapid tumor growth changes in the anti-PD-1 monotherapy group (Fig. 5f). IL-15 alone did not affect both CD8^+^ T cells and Tregs (Fig. 6a–c). This is possibly due to the limited number of target cells responding to IL-15 in tumors [32]. On the other hand, NIR-PIT reduced the number of Tregs (Fig. 6a), thus leading to a significant increase in the CD8^+^/Treg ratio in the Combination group. Even in the poorly-immunogenic model, the combination of NIR-PIT and IL-15 with anti-PD-1 mAb therapy demonstrated significant additional therapeutic effects that were not observed with anti-PD-1 mAb monotherapy (Fig. 5c, d). However, no CRs were seen in this model as would be expected (Fig. 5d). This is likely because of the low absolute number of immune cells, which is about 100-fold lower than the highly-immunogenic MC38-Luc cell line (Supplementary Fig. S7). Even when combined with anti-PD-1 mAb, the absolute number of CD8^+^ T cells was insufficient to mount a strong immune response. For poorly-immunogenic tumors, in addition to the therapies that act on the limited number of immune cells within the TME, it may be appropriate to further combine with tumor-targeted NIR-PIT that can fundamentally increase the absolute number of immune cells [33].

We acknowledge there is still room for optimization of treatment protocols. Due to the short half-life of IL-15, about one hour [34], we administered IL-15 multiple times. However, optimizing the frequency and the timing of administration (e.g., before or after PIT) may lead to a more effective therapeutic effect. Furthermore, the timing of anti-PD-1 mAb administration has not been optimized. In theory, anti-PD-1 mAb should be administered after augmenting the CD8^+^/Treg ratio with combination therapy. However, there was no established murine anti-PD-1 mAb administration protocol, so we used the same protocol as previously reported for the combination of NIR-PIT and anti-PD-1 mAb used in our previous study [12]. Consequently, we regard the protocol shown in Fig. 3a as a useful starting point for developing NIR-PIT therapeutic strategies against tumors lacking suitable target antigens because it demonstrated a high CR rate and high CD8^+^/Treg ratio.

In conclusion, the combination of CD25-targeted NIR-PIT and intratumoral IL-15 produces a favorable CD8^+^/Treg ratio, which improves the efficacy of anti-PD-1 mAb therapy, particularly in highly-immunogenic tumors. This combination therapy may prove useful in enhancing the effects of checkpoint immunotherapy.

## Supplementary Information

Below is the link to the electronic supplementary material.

Supplementary file1 (PDF 588 KB)

## Data Availability

The data supporting the findings of this study are available from the corresponding author upon reasonable request. All datasets were generated and analyzed using tumor-bearing mouse models. Due to the nature of animal experiments and institutional ethical regulations, raw data are not publicly deposited but can be shared for academic and non-commercial purposes upon request.

## Abbreviations

APC: Antibody-photo absorber conjugate
A.U.: Arbitrary units
CR: Complete response
DAPI: 4’,6-Diamidino-2-phenylindole
EGFR: Epidermal growth factor receptor
IL-15: Interleukin-15
IL-2: Interleukin-2
MC38-Luc: Luciferase-expressing MC38
mAb: Monoclonal antibody
NIR: Near-infrared
NK: Natural killer
PD-1: Programmed cell death protein 1
PIT: Photoimmunotherapy
TILs: Tumor-infiltrating lymphocytes
TME: Tumor microenvironment
Tregs: Regulatory T cells
