# Supplementary material for: Combined CD25-targeted near infrared photoimmunotherapy (NIR-PIT) and intratumor IL-15 enhance the effectiveness of anti-PD-1 immunotherapy
Source: Cancer Immunol Immunother. 2025 Dec 18;75(1):7. doi: 10.1007/s00262-025-04186-x (PMC12715065; doi:10.1007/s00262-025-04186-x)
Supplement: Supplementary file 1 — Supplementary file1 (PDF 588 KB) [file 262_2025_4186_MOESM1_ESM.pdf]

Electronic Supplementary Material

The supplementary figures will be submitted as a PDF.

Supplementary figures contain eight panels.

- Supplementary Figure S1 shows the body weight change in mice during treatment.
- Supplementary Figure S2 shows the gating strategy used to identify tumor-infiltrating $CD8^{+}$ T cells and Tregs by flow cytometry.
- Supplementary Figure S3 presents the population of natural killer T cells, dendritic cells and macrophages in MC38-Luc tumors.
- Supplementary Figure S4 displays representative fluorescent imaging at 700 nm in MC38-Luc.
- Supplementary Figure S5 contains the schedule and results of tumors rechallenge test.
- Supplementary Figure S6 displays treatment schedules and representative fluorescent imaging at 700 nm in MOC2 tumors.
- Supplementary Figure S7 presents the difference in immune cell number between MC38-Luc tumors and MOC2 tumors.

Supplementary Figure S1

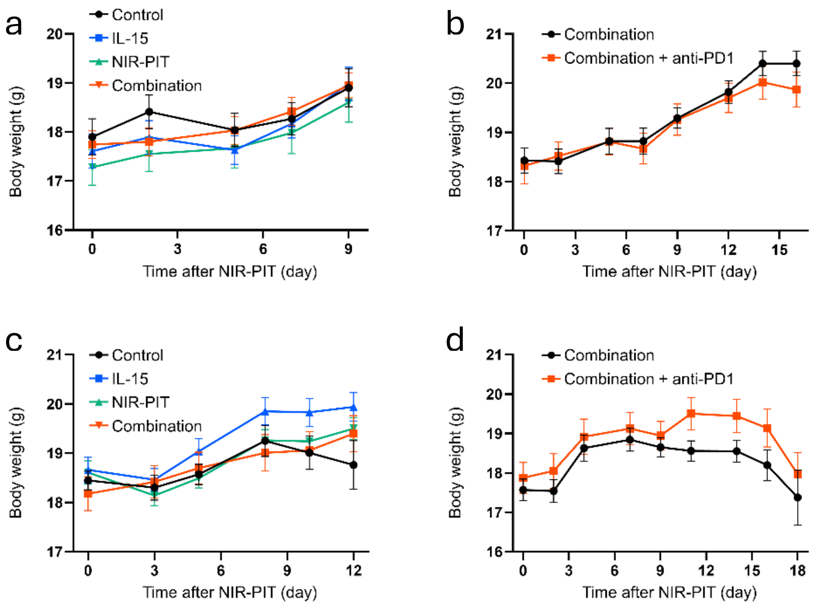

Body weight change during treatment

a, Body weight of MC38-Luc-bearing mice in comparison of intratumoral IL15 administration, CD25-targeted NIR-PIT, and their combination therapy (n = 10; mean ± SEM; repeated measures two-way ANOVA followed by Tukey's test); ns, not significant. **b**, Body weight of MC38-Luc-bearing mice in comparison of the combination therapy of intratumoral IL15 administration and CD25-targeted NIR-PIT, and their combination with anti-PD-1 mAb (n = 10; mean ± SEM; unpaired *t*-test on each day); ns, not significant. **c**, Body weight of MOC2-bearing mice in comparison of intratumoral IL15 administration, CD25-targeted NIR-PIT, and their combination therapy (n = 10; mean ± SEM; repeated measures two-way ANOVA followed by Tukey's test); ns, not significant. **d**, Body weight of MOC2-bearing mice in comparison of the combination therapy of intratumoral IL15 administration and CD25-targeted NIR-PIT, and their combination with anti-PD-1 mAb (n = 10; mean ± SEM; unpaired *t*-test on each day); ns, not significant.

Supplementary Figure S2

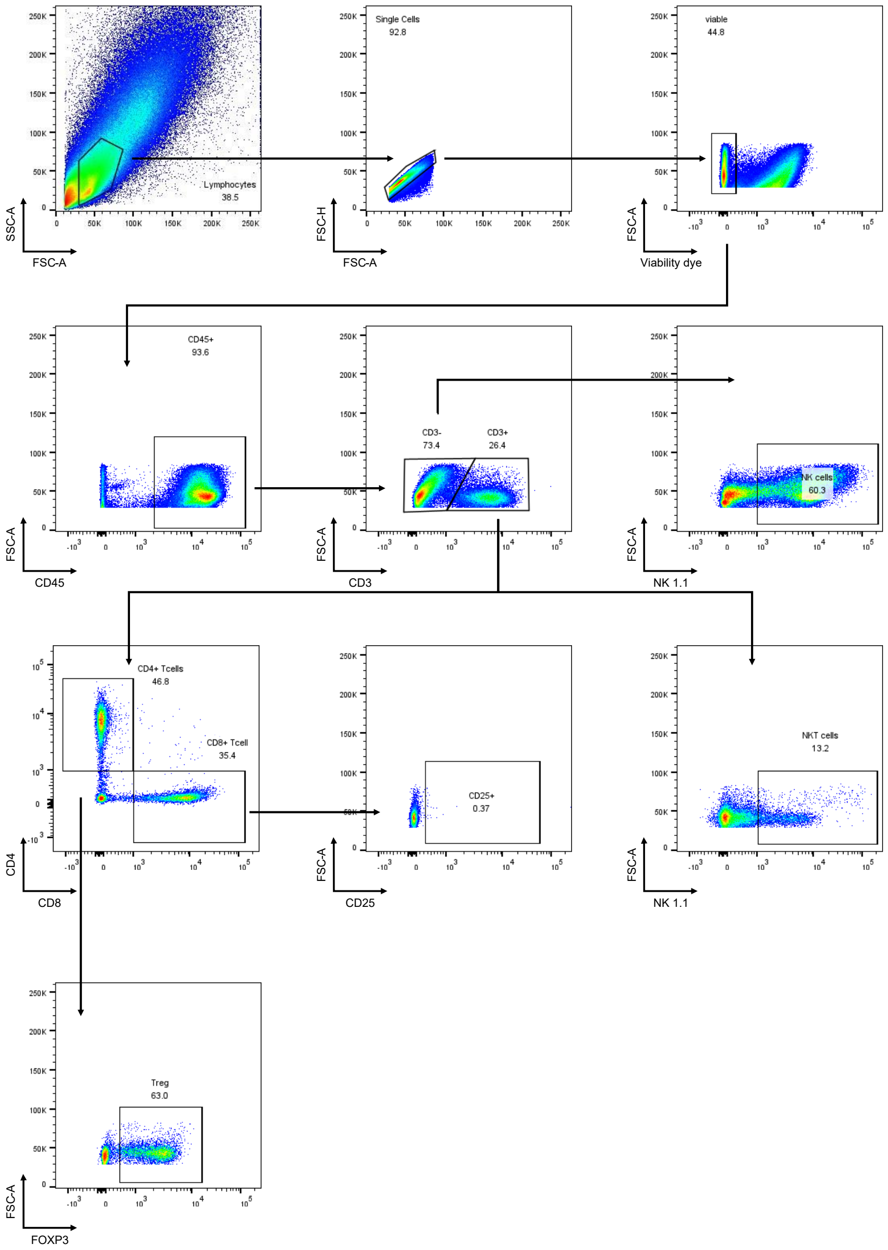

Flow cytometry analysis gating strategy

Cell types were determined as follows; $CD8^+$ T cells: $CD45^+/CD3^+/CD8^+/CD4^-$, regulatory T cells: $CD45^+/CD3^+/CD4^+/CD8^-/FOXP3^+$, Natural killer (NK) cells: $CD45^+/CD3^-/NK1.1^+$, and Natural killer T cells: $CD45^+/CD3^+/NK1.1^+$.

Supplementary Figure S3

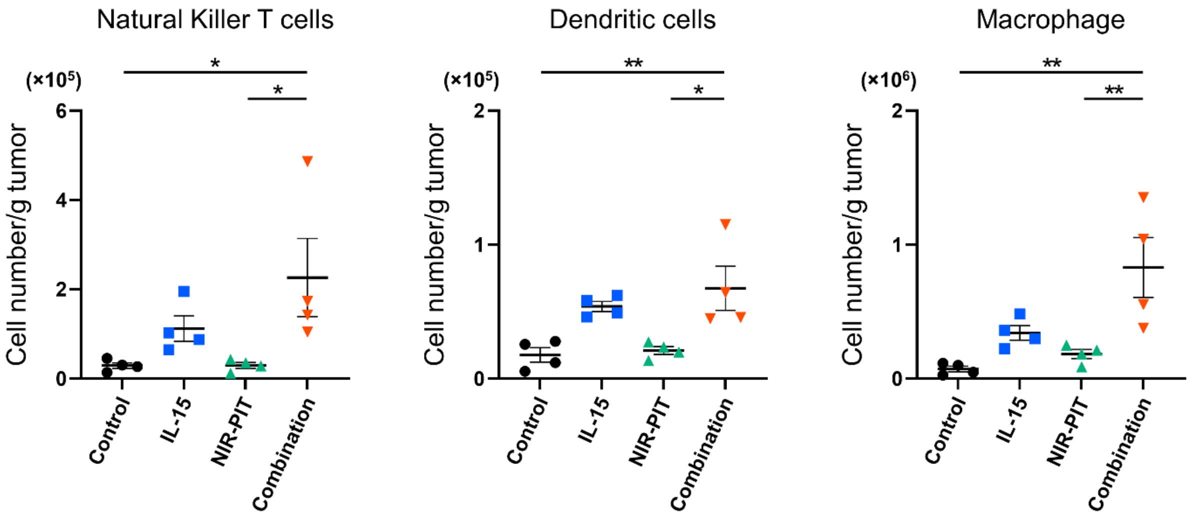

Immune cell populations within MC38-Luc tumor two days after CD25-targeted NIR-PIT

The cell number/g tumor of Natural killer T cells, Dendritic cells and Macrophages were evaluated by flow cytometry (n = 4; mean ± SEM; one-way ANOVA followed by Tukey test); *, $p < 0.05$; **, $p < 0.01$; multiple comparisons.

Supplementary Figure S4

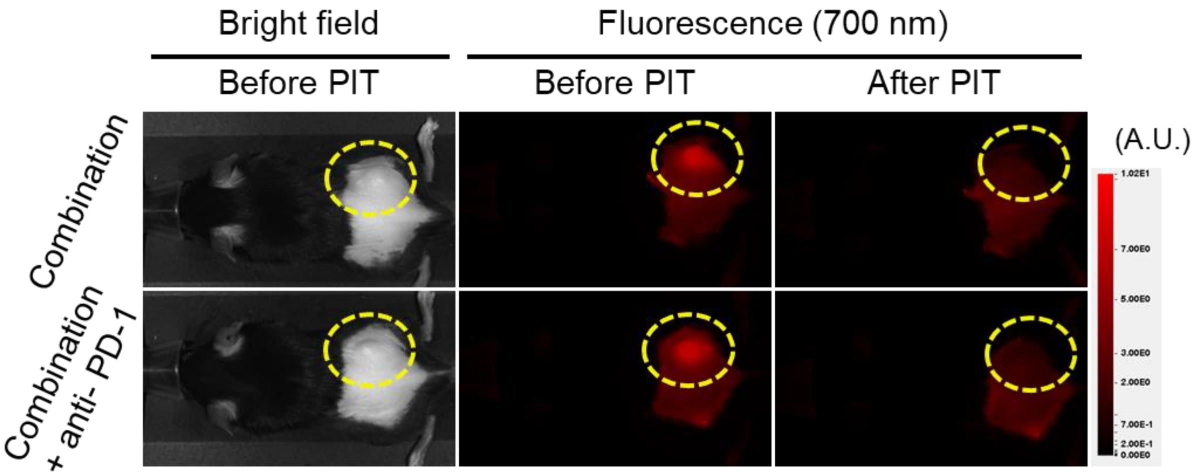

***In vivo* effect of anti-PD-1 mAb therapy on top of combination therapy of intratumoral IL-15 administration and CD25-targeted NIR-PIT for MC38-luc tumor mouse model.**

Representative fluorescent imaging at 700 nm before and after NIR-PIT. Left white images: Bright-field images. Right two red images: 700 nm fluorescence images. Yellow dashed circles surround the tumor sites.

Supplementary Figure S5

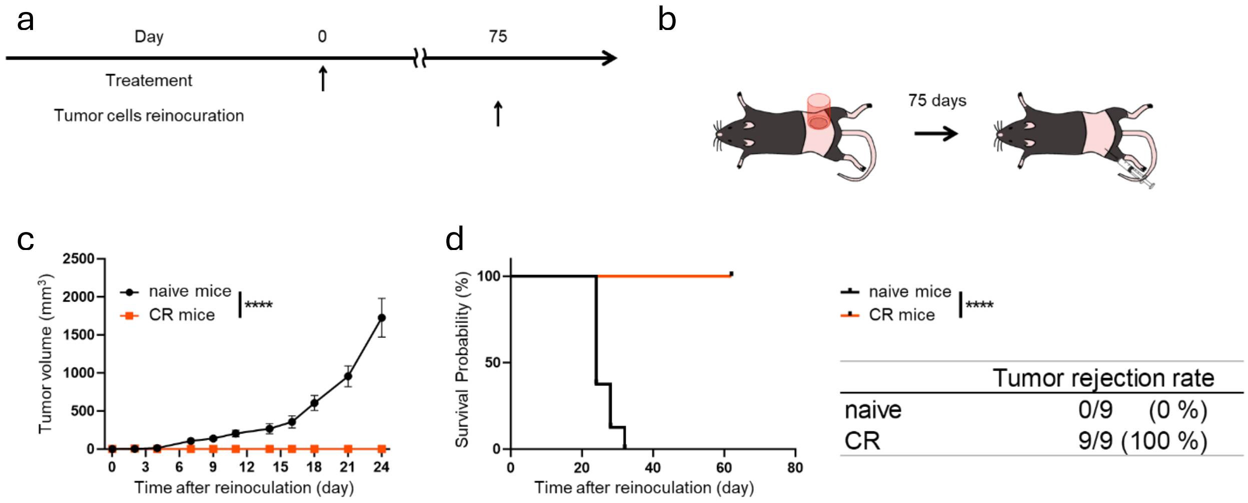

Tumors rechallenge test

a, Treatment schedule. MC38-Lucs were re-inoculated into nine mice that achieved a complete response, 75 days after the initial treatment. **b**, Diagram of this study. Reinoculation of tumor cells was performed on the contralateral flank. **c**, Tumor growth curves for anti-PD-1 mAb therapy group compared to control group (n = 9; mean ± SEM; repeated measures two-way ANOVA focusing on the group × time interaction); ****, $p < 0.0001$. **d**, Survival curves and summary table of tumor rejection rate (n = 9, log-rank test); ****, $p < 0.0001$.

Supplementary Figure S6

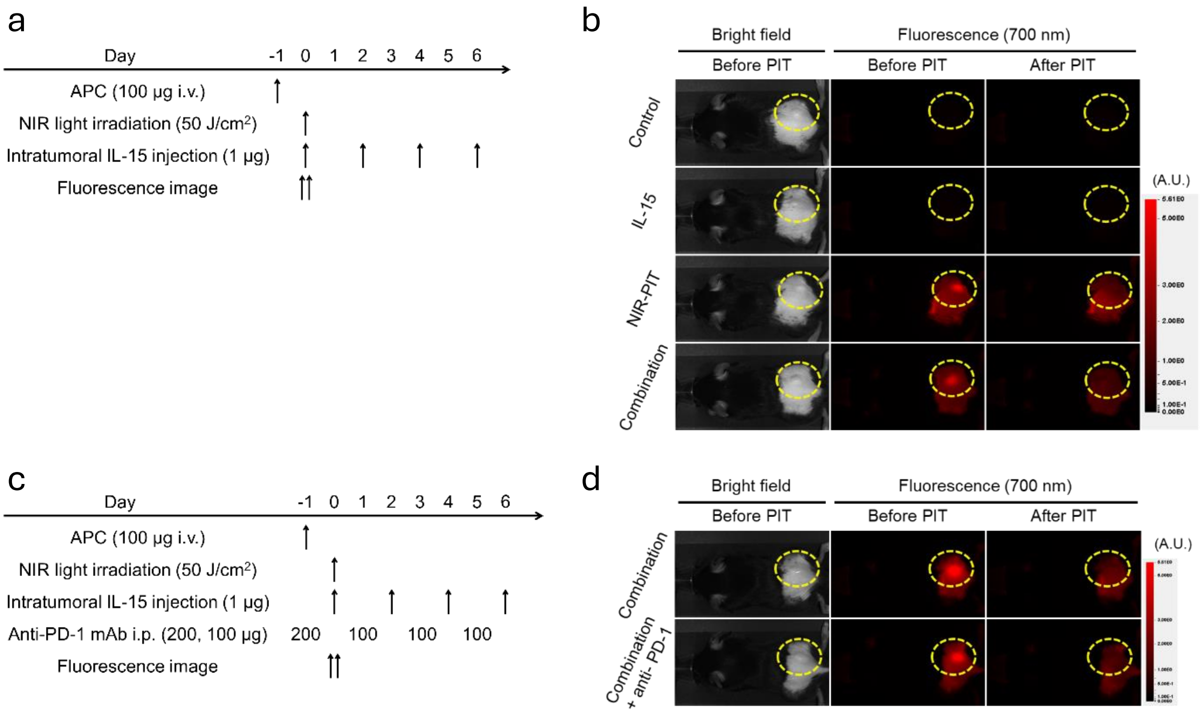

***In vivo* effect of intratumoral IL-15 administration and CD25-targeted NIR-PIT for MOC2 tumor mouse model**

a, Treatment schedule. **b**, Representative fluorescent imaging at 700 nm before and after NIR-PIT. Left white images: Bright-field images. Right two red images: 700 nm fluorescence images. Yellow dashed circles surround the tumor sites.

***In vivo* effect of anti-PD-1 mAb therapy on top of combination therapy of intratumoral IL-15 administration and CD25-targeted NIR-PIT for MOC2 tumor mouse model**

c, Treatment schedule. **d**, Representative fluorescent imaging at 700 nm before and after NIR-PIT. Left white images: Bright-field images. Right two red images: 700 nm fluorescence images. Yellow dashed circles surround the tumor sites.

Supplementary Figure S7

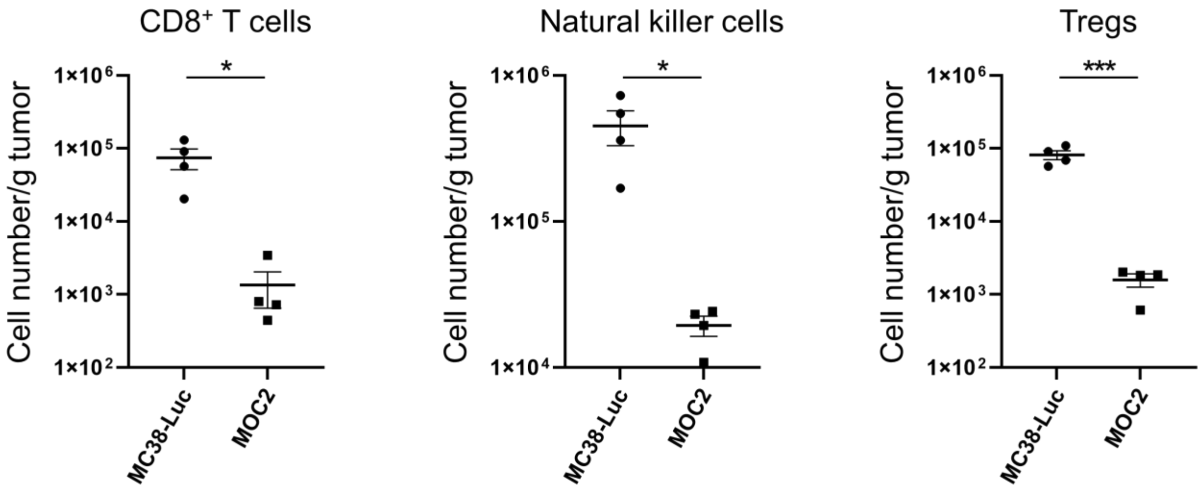

A comparison of immune cell populations between MC38-Luc tumor and MOC2

The cell number/g tumor of $CD8^+$ T cells, Natural killer cells, and Tregs was evaluated by flow cytometry. The Y-axis is shown on a logarithmic scale (note that zero values cannot be displayed; n = 4; mean ± SEM; unpaired *t*-test); *, $p < 0.05$; ***, $p < 0.001$.
